# *In silico* structural and docking models of dipteran FXPRLamide neuropeptides support ligand-receptor coevolution and suggest mechanisms for ligand bias

**DOI:** 10.1371/journal.pone.0329924

**Published:** 2025-12-29

**Authors:** Sarah M. Farris

**Affiliations:** Department of Biology, West Virginia University, Morgantown, West Virginia, United States of America; Indian Agricultural Research Institute, INDIA

## Abstract

Pyrokinin (PK) neuropeptides are characterized by a conserved C-terminal FXPRLamide motif and modulate a range of physiological functions and behaviors in species spanning the Eumetazoa. The insect pyrokinin gene *pban* encodes a taxon-specific number of PKs including the eponymous pheromone biosynthesis activating neuropeptide (PBAN). The *pban* gene of basal Diptera resembles that of other insect orders while in more derived Diptera (where it is referred to as the *hugin* gene) the PBAN peptide coding sequence appears to be absent. In the present study, *in silico* structural models and docking simulations of the *Drosophila melanogaster* PK hugin and its receptor PK2-R1 are compared with those of the PBAN and PK2–3 neuropeptides (the latter the likely homolog of hugin) and their receptor PK2/PBAN-R belonging to the more basal species *Aedes aegypti*. The binding pockets for all three peptides overlap extensively as do individual amino acid contacts; these interactions also concur with data for PBAN and NMU binding of their cognate receptors in the silk moth *Bombyx mori* and humans respectively. C-terminal FXPRL core sequences of all peptides preferentially bind conserved residues in the transmembrane regions (TMs) of the receptor while the variable N-termini interact with amino acids in the extracellular loops (ECLs) that differ between the two species. The *A. aeg* PBAN peptide forms rigid secondary and tertiary structures with its long N-terminus that uniquely interact with non-conserved amino acids in the extended ECLs of PK2/PBAN-R, providing a basis for functional differentiation from binding of the short and flexible PK2–3 peptide to the same receptor, perhaps via a mechanism such as ligand bias. *D. melanogaster* hugin is similar in structure to PK2–3 but interacts with amino acids in areas of PK2-R1 that contact PBAN but not PK2–3 in *A. aegypti,* even though amino acids at those receptor sites are non-synonymous substitutions compared with PK2/PBAN-R. The ECLs of PK2-R1 are also shorter than those of PK2/PBAN-R, reflecting the loss of selection for contacts with the much longer PBAN peptide. Taken together these changes suggest that loss of PBAN impacted ligand-receptor coevolution in the higher Diptera.

## Introduction

Neuropeptides are evolutionarily ancient signaling molecules that function as juxtacrine, paracrine, or endocrine modulators of physiology and behavior. Many neuropeptide families and their G protein-coupled receptors (GPCRs) are conserved across the Metazoa, underscoring the importance of neuropeptide signaling [1,2]. However, diversification and coevolution of neuropeptides and their cognate receptors reflect their adaptability to the physiological and behavioral requirements of diverse ecological niches encountered over millions of years of animal evolution [3–5].

### The PRXamide family of neuropeptides

Genes encoding PRXamides have been identified in the genomes of animals across the Metazoa based on their shared C-terminal sequence, in which the terminal “X” is typically an uncharged residue (for review see [6]). Within the protostomes, PRXamides and their receptors have been recovered from species representing the Euarthropoda, Mollusca, Annelida, Nematoda and the Cnidaria. They are considered homologous to deuterostome neuromedin U, with more distant relationships to TRH and ghrelin [5,7–12]. In the Panarthropoda, genes encoding three classes of PRXamides have been identified: *ecdysis-triggering hormone* (*eth*), *capability* (*capa*), and pheromone biosynthesis-activating neuropeptide (*pban*, or *hugin* in *Drosophila melanogaster*; for review see [6]). ETH peptides regulate ecdysis and are conserved across the Panarthropoda; unlike the other PRXamides they are produced by secretory cells of the epitracheal glands and are thus not neuropeptides [13]. *Capa* and *pban* genes have been isolated from species of Chelicerata and Pancrustacea. *Capa* encodes a taxon-variable number of periviscerokinins (PVKs) that have myotropic and osmoregulatory functions, and a diapause hormone (DH) that regulates diapause onset [14–20]. In insects the *pban* gene encodes the eponymous pheromone biosynthesis activating neuropeptide PBAN and two or three additional PK2s, together referred to as PK2/PBANs; in some species a diapause hormone with a WFGPRLamide C-terminal sequence, termed PK1/DH, may also be encoded by the *pban* gene [20–35]. The PK2s are inconsistently named in the literature but this account will use the terms PK2–1, PK2–2, PBAN, and PK2–3 in order from the N-terminus to C-terminus of the prepropeptide according to nomenclature utilized in [36]. PK2–1 appears to be restricted to the Lepidoptera while PK2–2, PBAN and PK2–3 peptides are present in many species (for reviews see [36–38]). All pyrokinins bind Class A G protein-coupled receptors (GPCRs) with separate receptors for PK1/DH (PK1/DH-R) and for PK2/PBANs (PK2/PBAN-R), although in some species PK1/DH activates PK2/PBAN-R and vice versa albeit with lower affinities [22,26,31,33,39–48]. Additional diversity of cellular responses is provided by tissue-specific expression of multiple receptor splice isoforms as in Lepidoptera or duplication of the receptor as observed in the higher Diptera and the Coleoptera [43–45,49,50].

### Roles of neuropeptides encoded by the PK/PBAN gene

As the name suggests, PBAN is a regulator of sex pheromone biosynthesis and/or release [27,30,51–53] although functional divergence is apparent in other species. Supporting a function as a general regulator of intraspecific communication, PK2/PBAN neuropeptides initiate aggregation pheromone synthesis in the western flower thrips *Frankliniella occidentalis* [25] and trail pheromone synthesis in the fire ant *Solenopsis invicta* [29]. The trail pheromone gland is derived from Dufour’s gland [54], which produces several intraspecific communication signals including sex pheromone in Hymenoptera [55,56]. Intriguingly, transformation of an entomopathogenic fungus with the *Solenopsis invicta* coding sequence for PK2–2 increased virulence of a fungal insecticide and behavioral abnormalities in the ant [57]. Additional roles for peptides encoded by the *pban* gene include regulating cuticle pigmentation [40,58] and timing of life history transitions such as pupariation and diapause [14,59–61]. In the fruit fly *Drosophila melanogaster* the *pban* gene homolog *hugin* encodes two peptides, hugin and γ-hugin [28]. γ-hugin is likely homologous to PK2–2 and hugin to PK2–3 based on their length, sequence conservation and location within the prepropeptide [62]. Hugin has been shown to play complex roles in the regulation of feeding-associated locomotor behavior under circadian regulation in *Drosophila melanogaster* [63–67]. The functions of the remaining PK2 peptides encoded by the *pban* and *hugin* genes are poorly understood although functional studies reveal that in many cases these peptides bind and activate PK2/PBAN receptors [22,31,39–42,45–48,68].

### Pyrokinin neuropeptides and their receptors in Diptera

The *pban* gene in the basal Diptera encodes a prepropeptide with an ancestral complement of neuropeptides: PK1/DH, PK2–2, PBAN and PK2–3 [21,62]. PK1/DH is lost at the origin of the Neodiptera and PBAN is absent in the Syrphidae + Schizophora based on the loss of the FXPRL motif [62]. Variable γ-hugin and conserved SVXFKPRL (hugin) sequences are characteristic of the Brachycera [62]. In *D. melanogaster* γ-hugin possesses a canonical FXPRLamide motif and activates the *D. melanogaster* PK2/PBAN-R (termed PK2-R1) *in vitro* even though the peptide is undetectable *in vivo* [26,28] while hugin has a diverse array of functions as mentioned above.

### Ligand-receptor coevolution of pyrokinins in Diptera

It is well established that conserved neuropeptide families coevolve with their receptors [4,31,50,69]. For example, sequence and concomitant structural divergence of the PRXamides PVK, PK1/DH and PK2/PBANs and their receptors means that each neuropeptide binds its cognate receptor with high affinity but the other PRXamide receptors with lower affinities or not at all as shown in functional studies in several insect species [22,26,31,33,39–48]. However, structural models of FXPRLamide ligand-receptor interactions are limited to just two studies restricted to the conserved C-terminal FSPRLamide (*Bombyx mori*, [70]) or YFSPRLamide (*Helicoverpa zea*, [71]); the role of the variable N-terminus of FXPRLamides in receptor binding is poorly understood. This study employs *in silico* structural models and ligand-receptor docking simulations to determine whether loss of the PBAN neuropeptide in the higher Diptera is associated with corresponding changes in PK2-R1 and whether binding interactions characteristic of PK2–3 binding in basal Diptera are conserved due to retention of the homologous hugin peptide. In doing so, this study reveals highly conserved receptor binding pockets containing both synonymous and non-synonymous amino acid binding sites for each peptide. Additionally, PBAN and PK2–3/hugin peptides are characterized by distinct secondary and tertiary structures that confer different constraints on receptor binding. Taken together these findings suggest a potential basis for differential modulation of PK2/PBAN-R activation by PK2–3 and PBAN in *A. aegypti,* perhaps by a mechanism such as ligand bias, and coevolution of hugin and PK2-R1 in *D. melanogaster*, all while maintaining recognition of the FXPRL core motif.

## Methods

### Gene sequences and binding pockets

The translated *Aedes aegypti* PBAN (GenBank accession #XM_001662162 and PK2/PBAN-R (GenBank accession #KC155994) [21] and *Drosophila melanogaster* hugin (CG6371, accession #AJ133105) [11,28,72] and PK2-R1 (CG8784, accession #AF522189) [45,50] were aligned using Clustal Omega [73]. The *D. melanogaster* genome also contains a second PK2 receptor (PK2-R2, CG8795) with 58.8% identity to CG8784. Both receptors are strongly activated by hugin [45] (although also see [50]), but only PK2-R1 has been shown to mediate physiological functions of hugin in *D. melanogaster* [74] and was thus chosen for investigation in this study. Binding pockets of each receptor were identified from sequence data using PrankWeb [75], and the top-ranked prediction was mapped onto sequence alignments. Predicted amino acid contacts between ligand and receptor, using the methods described in the following section, were also mapped onto sequence alignments and onto receptor secondary structural models generated using the Protter tool [76].

### Modeling peptide-receptor binding

Receptor structure models were retrieved from the AlphaFold protein structure database (*A. aegypti* PK2/PBAN-R AF-V9P4J1-F1-v4; *D. melanogaster* PK2-R1 AF-Q9VFW5-F1-v4) [77]. The flexible, disordered extracellular N- termini of PK2/PBAN-R up to residue 79 and PK2-R1 to residue 96 were removed for docking simulations (see Discussion for rationale). Structure models of each peptide were generated from the active peptide sequences (*A. aegypti* PBAN: DASSSNENNSRPPFAPRL; PK2–3: NLPFSPRL and *D. melanogaster* hugin: SVPFKPRL using PepFold4 [78]. Only those peptide models that formed a β-turn between the X and L residues of the core sequence, required for receptor activation, were selected for docking analysis [79–81]. The C-terminal α-amidation was not included in peptide models (see Discussion for rationale. Two tools were employed to assess interactions between individual amino acids of peptides and receptors. MDockPep generated docking predictions using receptor structural models and peptide sequences [82]. Valid models of the full PBAN neuropeptide (those that contained the C-terminal β-turn) could not be produced with the MDockPep tool, so models of the truncated C-terminal RPPFAPRL peptide were used instead. Predictions made from structural models of both peptides and receptors were generated using ClusPro 2.0 [83]. Only those models in which the C-terminal peptide core sequence penetrated the binding pocket and that were within the top 10 predictions for each valid peptide as described above were selected for further investigation. The interactions between ligand and receptor amino acids were largely overlapping amongst models for each peptide and receptor and were combined for the final determination of docking predictions. Predicted ligand-binding domains were mapped onto receptor sequences using Clustal Omega and Protter as described above. Docked peptides and receptors were imported into UCSF ChimeraX [84] for visualization, identification of amino acid contacts, presence and location of peptide β-turn hydrogen bonds and matchmaking between isolated and full peptide core structures.

## Results

### Receptor binding pocket and ligand docking predictions for *A. aegypti* PBAN and PK2–3 and *D. melanogaster* hugin and their cognate receptors PK2/PBAN-R and PK2-R1

**Fig 1:**
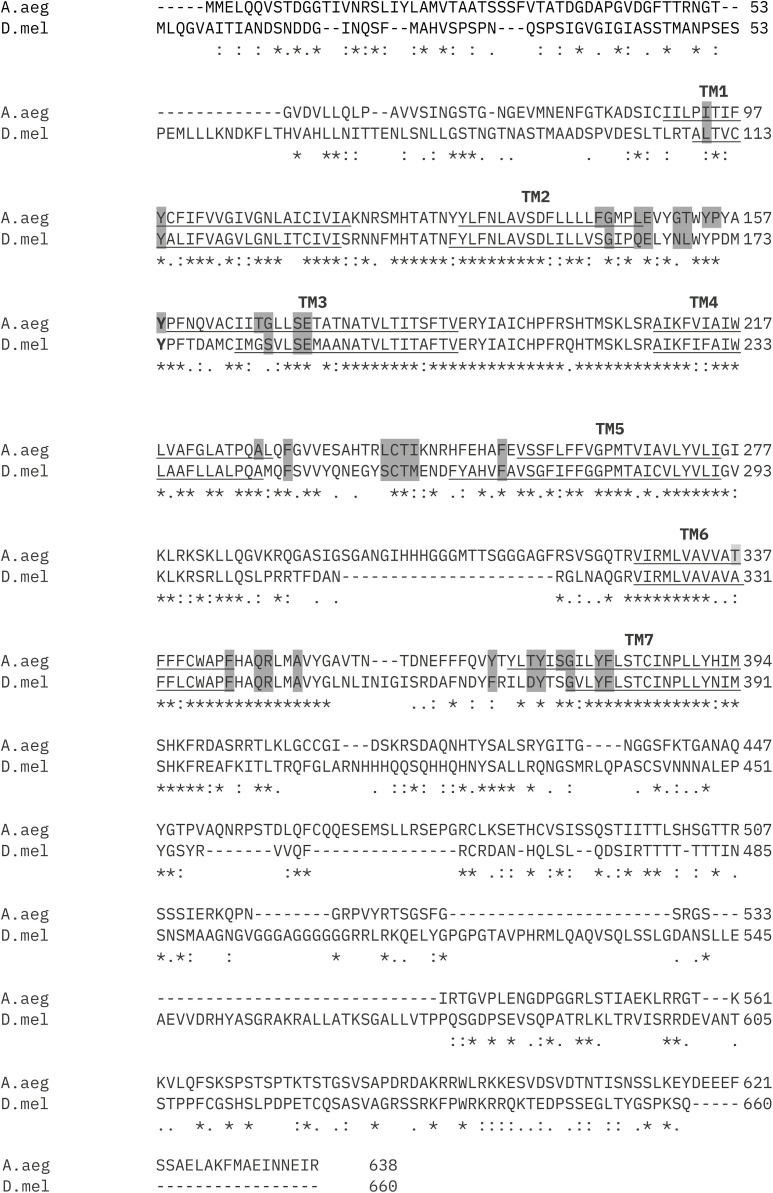
Predicted binding pockets in *A. aegypti* PK2/PBAN-R and *Drosophila melanogaster* PK2-R1. Transmembrane regions are underlined and the top-ranked binding pocket residues predicted by PrankWeb are indicated in dark grey. Annotations below alignment: * fully conserved,: strongly conservative substitution,. weakly conservative substitution, no symbol non-conserved substitution.

Sequence alignment of *A. aegypti* PK2/PBAN-R and *D. melanogaster* PK2-R1 proteins revealed substantial homology with an overall identity of 43.49% (Fig 1, S1 Table). The lowest identity occurs in the extracellular N-terminus (18.3%), the intracellular C-terminus (23.4%), and the third intracellular loop (ICL3; 34.5%)**.** The second intracellular loop (ICL2; 95%) and transmembrane domains (TMs) 4–7 (71.43–79.17%) are most highly conserved, consistent with their known roles in G protein coupling and signal transduction [85–89]. Binding specificity of peptide ligands to their cognate GPCRs occurs mostly via interactions with the three extracellular loops (ECLs) and extracellular-facing residues of the transmembrane domains [88,90–92]; these regions have low to intermediate similarity between the two species. Binding pocket predictions made using PrankWeb corroborate these features of PK2/PBAN-R and PK2-R1.

**Fig 2:**
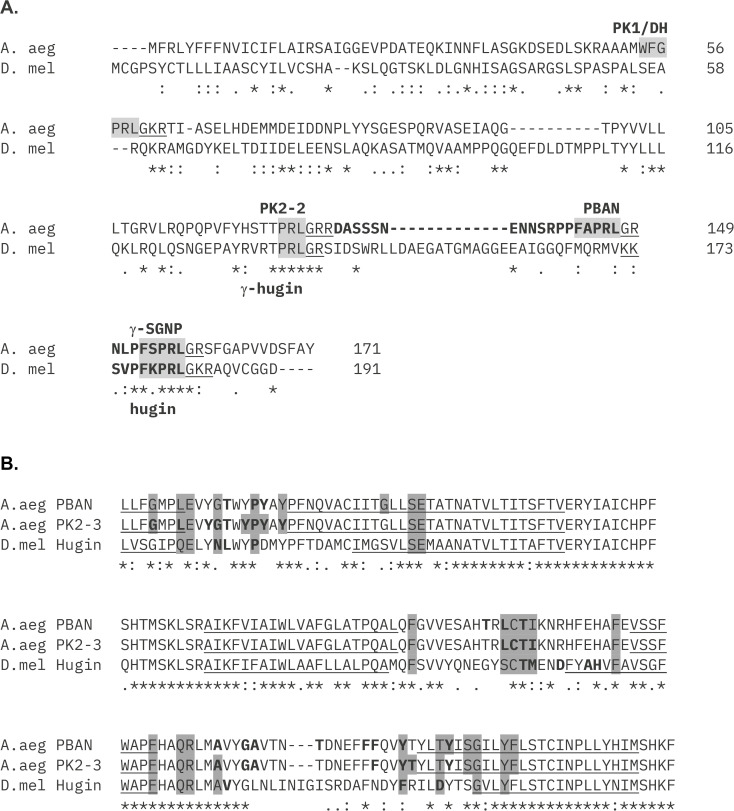
Sequences of *pban-* and *hugin*-encoded prepropeptides and ligand-receptor residue contacts as determined by MDockPep. **A.** *A. aegypti* PBAN and *D. melanogaster* hugin prepropeptides. PBAN, PK2-3 (Q16N80) and hugin (NP_524329) peptide sequences are bolded and labeled. Core motifs are indicated by a shaded box while amidation and peptidase cleavage sites are underlined. Locations of core motifs of additional pyrokinin peptides are labeled and highlighted in grey. **B.** Peptide binding residues of *A. aegypti* PK2/PBAN-R (accession number AGT80483) and *D. melanogaster* PK2-R1 (NP_731790) as predicted using peptide sequence data and receptor structural models by MDockPep. Grey boxes indicate core sequence binding (FXPRL), bold indicates binding by N-terminal residues of the peptide, both indicate binding by core and N-terminal residues. Only extracellular loops and nearby transmembrane regions (underlined) are shown. Annotations below alignment: * fully conserved,: strongly conservative substitution,. weakly -conservative substitution, no symbol- = non-conserved substitution.

Sequences of the *A. aegypti* PBAN and PK2–3 and *D. melanogaster* hugin prepropeptides are shown in Fig 2A. Active neuropeptides are α-amidated at the C-terminal glycine (G) residue preceding dibasic cleavage sites (underlined). The active PBAN peptide of *A. aegypti* is characterized by a FAPRL core motif (shaded) and a long N-terminal sequence (DASSSNENNSRPP); PK2–3 consists of a FSPRL core and a short N-terminus (NLP). Hugin, like PK2–3, has a three amino acid N-terminus (SVP) but an FKPRL core motif. MDockPep predictions of interactions between individual peptide amino acids with those of the receptor (Fig 2B) are consistent with binding pocket predictions in Fig 1. Neither PrankWeb nor MDockPep predicted ligand interactions with receptor N-termini, in contrast with docking predictions generated using ClusPro (see below). Receptor binding by the FXPRL core sequence (shaded) is frequently localized to the transmembrane regions (underlined) while binding sites of the peptide N-termini (bold) often occur in the ECLs and receptor N-terminus, although there is substantial overlap between the two (shaded + bold). Overall ligand-receptor interactions between the dipteran peptides and receptors, *Bombyx mori* PBAN and PK2/PBAN-R, and human NMU/NMS peptides with NMUR1 and NMUR2 (the vertebrate homologs of insect pyrokinins/PKRs) reveal similar conservation of binding sites especially in the ECLs, TM3 and TM7 [70,93,94] (S1 Fig).

**Fig 3:**
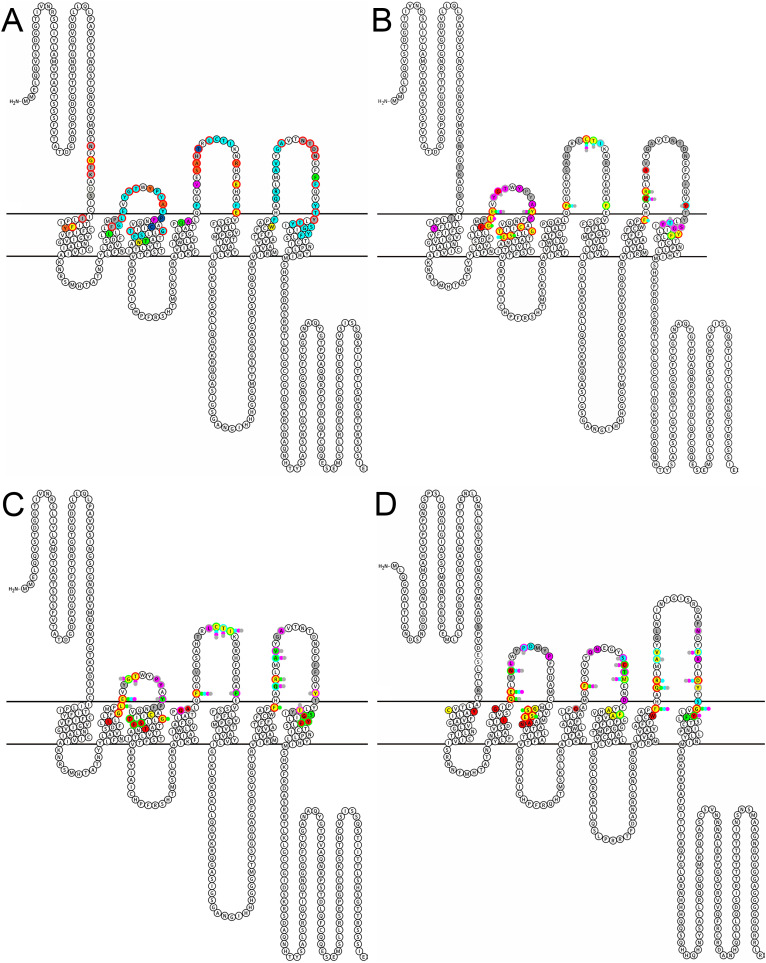
Combined ligand-receptor amino acid contacts determined by Clus-Pro molecular docking predictions. **A.** Summary of all possible *A. aegypti* PBAN, PK2-3, and *D. melanogaster* hugin peptide binding sites gleaned from all valid docking predictions using the *A. aegypti* PK2/PBAN-R as a template. Light blue, receptor residue bound by all peptides; grey, bound only by *A. aegypti* PBAN; magenta, bound only by *A. aegypti* PK2-3; dark blue, bound by both *A. aegypti* PBAN and PK2-3; yellow, bound only by *D. melanogaster* hugin; green, bound by both *A. aegypti* PK2-3 and *D. melanogaster* hugin; orange- bound by both *A. aegypti* PBAN and *D. melanogaster* hugin. Red circles- weakly conservative or non-conservative mutation between *A. aegypti* and *D. melanogaster*. B-D. Binding of peptide ligands to their cognate receptor by individual peptide residue. B. *A. aegypti* PBAN:PK2/PBAN-R. C. *A. aegypti* PK2-3:PK2/PBAN-R. D. *D. melanogaster* hugin:PK2-R1. Grey, N-terminal peptide amino acids: FXPRL residues magenta, Phe (F); blue, X; green, Pro(P); red, Arg (R); yellow, Leu (L).

ClusPro docking predictions using structural models of the peptides and their receptors revealed substantial overlap of ligand-receptor binding patterns overlaid by peptide- and species-dependent variation. The snake plot in Fig 3A summarizes amino acid contacts made by all valid ligand-receptor docking poses of the three peptides studied here, using PK2/PBAN-R of *A. aegypti* as the template. Thirty-five receptor amino acids interact with all three ligands (light blue), with some engaging the same amino acids in all ligands of both species (S2 Fig) even if a non-synonymous amino acid substitution occurs between *A. aegypti* and *D. melanogaster* (red outline). This exceeds the number of receptor contacts predicted by MDockPep, reflecting multiple valid structural models and docking predictions generated by PepFold4 and ClusPro that were combined for this figure. *A. aegypti* PBAN has the most unique receptor binding interactions (13 residues; Fig 3A grey), primarily involving non-synonymous sites in the ECLs. Hugin makes six unique interactions (yellow), four of which are with non-synonymous residues, and PK2–3 makes three unique interactions with conserved receptor residues (magenta). Hugin shares six binding sites with PBAN (orange; four non-synonymous) and three with PK2–3 (green; all conserved).

Analysis of specific ligand-receptor interactions using Chimera X to identify contacts in ClusPro-generated docking models reveals consistent features across peptides and species (Fig 3B–3D; S2 Table). Concordant with the MDockPep data, individual neuropeptide residues bind multiple receptor residues, and vice versa. As previously described, FXPRL core motifs, especially the proline-arginine-leucine residues (PRL; green, red and yellow respectively) of all peptides interact frequently with transmembrane domains. Phenylalanine (F) and X residues (magenta and blue) of the core motif often share binding sites in the ECLs with the N-terminal domains of the peptides. Interactions between peptide N-termini (grey) and distal ECLs more frequently involve a single peptide residue with the exception of a cysteine-containing- β-turn motif in ECL2 characteristic of GPCRs that binds nearly all core motif and N-terminal peptide residues [81]. In all peptides the variable “X” of the FXPRL core sequence forms the fewest bonds with the receptor and is almost entirely restricted to the ECLs (Fig 3B–3D; S2 Table). The core motif proline residue (P) of PBAN and hugin also binds infrequently but in PK2–3 makes additional contacts in TM3 and ECL3. The core motif C-terminal leucine (L) of hugin interacts more frequently with ECL3 than is observed for the other peptides. The PBAN N-terminus binds PK2/PBAN-R almost exclusively at the ECLs and receptor N-terminus and has the most total binding sites, in keeping with the long (13 amino acid) N-terminus compared with those of the other two peptides (3 amino acids each).

### Molecular structural models of FXPRL neuropeptides and docking simulations

**Fig 4:**
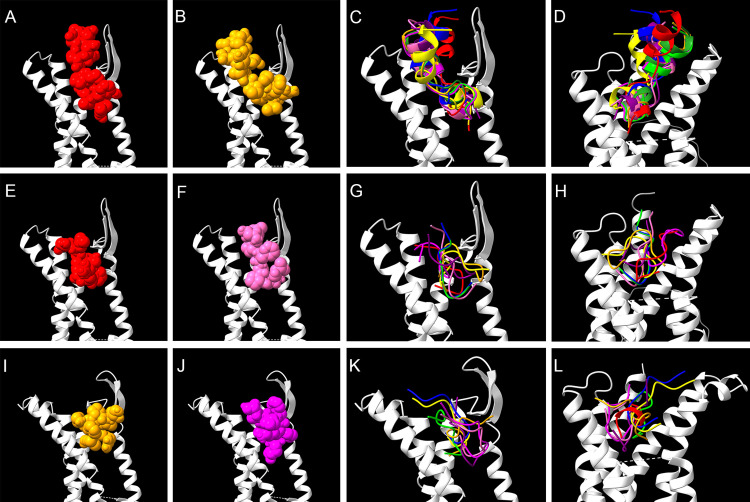
Docking predictions using peptide and receptor structural models. A-B: Top two docking predictions for *A. aegypti* PBAN to PK2/PBAN-R. ECL1 and N-terminus are removed to provide a clearer view of the peptide. C: Ribbon models of all valid PBAN peptide structural models and docking predictions with the N-terminus and ECL1 removed. D: Same as previous except with ECL2 removed. E-F: Top two docking predictions for *A. aegypti* PK2-3 to PK2/PBAN-R. ECL1 and N-terminus are removed to provide a clearer view of the peptide. G: all valid PK2-3 peptide docking predictions with the N-terminus and ECL1 removed. H: Same as previous except with ECL2 removed. I-J: Top two docking predictions for *D. melanogaster* hugin to PK2-R1. ECL1 and N-terminus are removed to provide a clearer view of the peptide. K: All valid hugin peptide docking predictions with the N terminus and ECL1 removed. L: Same as previous except with ECL2 removed.

Docking predictions generated by ClusPro for PBAN, PK2–3 and hugin revealed differences in secondary and tertiary peptide structures and their interactions with their cognate receptors (Fig 4, S3 Fig). Fig 4A–4B, 4E–4F, and 4I–4J depict the top two ClusPro predictions using the top two PepFold4 models of PBAN, PK2–3 and hugin respectively. The figures illustrate the greater length of the PBAN peptide allowing interaction with distal portions of the ECLs in comparison with the compact PK2–3 and hugin peptides. Ribbon models in Fig 4C–4D, 4G–4H and 4K–4L combine all valid molecular models for each peptide. There is variability among the models, especially PK2–3 and hugin. Orientation of the core motif β-turn in the binding pocket among the viable models of all three peptides also varies. PBAN is unusual in that its extended N-terminus forms a rigid helix that rests alongside ECL3 and approaches ECL2 (Fig 4C, 4D). The FAPRL core also adopts a helical structure, consistent with the helix-promoting and stabilizing properties of alanine (A) and proline (P) [95,96]. PepFold4 models of PK2–3 reveal less ordered structures, and docking predictions suggest that peptide orientation within the binding pocket is more flexible that observed for PBAN **(**Fig 4E–4H**)**. The short N-terminus remains within the binding pocket but is also predicted to be capable of a wider range of receptor interactions than for PBAN. Hugin models and predictions display similar features to PK2–3 (Fig 4I–4L).

**Fig 5:**
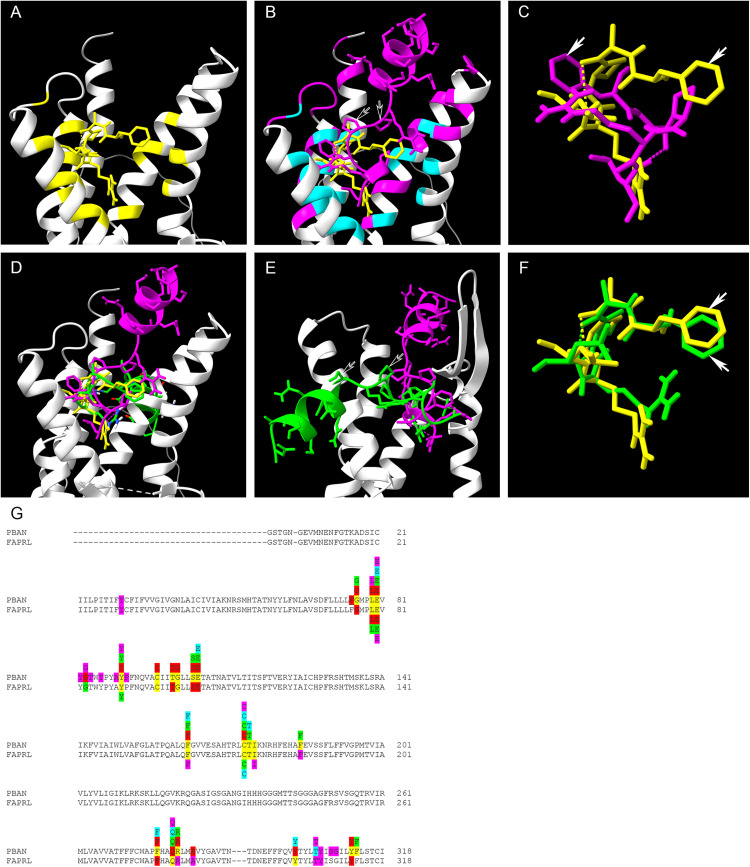
Docking of the PBAN core motif and the full *A. aegypti* PBAN peptide to PK2/PBAN-R before and after alignment of core sequences. **A.** Receptor residues contacted by the isolated FAPRL core sequence (yellow), with ECL2 removed for clarity. **B.** Isolated core motif (yellow) and full PBAN peptide (magenta) docked to PK2/PBAN-R. Overlap of receptor contacts between the isolated core motif and the full peptide are indicated in blue, PBAN peptide N-terminal contacts only in magenta. Arrows- proline residues producing a “proline kink” in the full peptide. **C.** Overlap of isolated (yellow) and full peptide (magenta) core motif docking predictions (arrows). Arrows- phenylalanine residues. **D.** Docking of the isolated core motif (yellow) and full PBAN peptide before (magenta) and after (green) after forced alignment of the isolated core motif with that of the peptide. The helical N-terminal of the PBAN peptide is oriented orthogonally with respect to the ECLs, through the back of the page. **E.** Same as panel D but with the receptor rotated 180 degrees and ECL1 removed for a better view of the altered position of the peptide N-terminus. Double prolines (arrows) introduce a kink in the backbone that directs the helical N-terminus orthogonal to the transmembrane regions and through ECL3. **F.** Correspondence of the isolated core motif (yellow) and that of the full peptide (green) after forced alignment. Arrows- phenylalanine residues. **G.** Consensus of docking predictions for the isolated FAPRL and full PBAN core motifs binding PK2/PBAN-R, with colors indicating residues of each peptide. Magenta, Phe (F); blue, Ala (A); green, Pro (P); red, Arg (R); yellow, Leu (L); grey, N terminus (full length peptide only). Only receptor ECL and TM binding regions are shown.

The isolated FAPRL core sequence of PBAN interacts with the same receptor residues (yellow, Fig 5A, 5B) as observed for the full peptide (overlap indicated in blue, Fig 5B). Arrows indicate two proline residues between the core motif and helical N-terminus in the full peptide that introduce a “proline kink” in the peptide backbone that directs the N-terminus of the full peptide (magenta) through the center of the receptor. Structural models of bound isolated core sequences (yellow) are not aligned with the full peptide core sequence (magenta, Fig 5C). Forced alignment of the full peptide and isolated core sequences using the Matchmaker function in Chimera X dramatically reorients the full PBAN N-terminus so that instead of projecting through the center of the binding pocket it is directed orthogonally by the proline kink, clashing with ECL3, TM6 and TM7, (green Fig 5D, 5E; arrows in the latter indicate proline residues). Forced alignment of the isolated (yellow) and full peptide core sequence (magenta) moves the phenylalanine (F) residue of the latter to nearly the same position as in the isolated core (Fig 5F, arrows). Docking predictions for the isolated core sequence would thus result in impermissible orientations of the full peptide as shown in Fig 5D and 5E. Fig 5G depicts a consensus alignment of peptide of full PBAN and isolated core motif binding to PK2/PBAN-R, color coded by peptide residue. While the overall pattern of interactions is similar, individual amino acids of the full peptide core sequences make more frequent contacts that often bind more than one peptide residue. The distribution of binding sites across the receptor also differs. For example, F of the PBAN FAPRL core motif is adjacent to ECL1 in the full peptide but more centrally positioned in the isolated core sequence, resulting in fewer interactions between F and ECL1 in the latter. Similar analyses were conducted for PK2–3 and hugin (S4 Fig). As with PBAN, these peptides have a proline residue immediately before the core sequence, with the resulting kink directing the N-terminus along the inner surface of ECL3 but orthogonally through the ECL when isolated and full peptide core sequences are forced to align.

## Discussion

### Binding pockets and ligand-receptor residue interactions in Diptera reflect conserved features of PRXamides and Class A GPCRs across Metazoa

This study compares intraspecific interactions between PBAN and PK2–3 with their receptor PK2/PBAN-R in *A. aegypti*, and interspecific differences between these and hugin (PK2–3 homolog) and its receptor PK2-R1 in *D. melanogaster*. Within species, PK2/PBAN-R binds promiscuously to *pban* gene-encoded peptides with varying affinities [22,26,31,33,39–48]. The data presented here provide insight into sequence and structural features of each peptide that confer unique binding interactions with PK2/PBAN-R that might enable peptide-specific functions in *A. aegypti*. Coevolutionary changes between *D. melanogaster* hugin and PK2-R1, including non-synonymous changes in receptor amino acids at binding sites shared across all peptides, acquisition of binding sites occupied by PBAN in *A. aegypti*, and fixation of the SVPFKPRL hugin sequence are observed. The novel roles in circadian regulation of feeding and locomotor behaviors that have been uncovered in *D. melanogaster* may have evolved concurrently with such coevolutionary changes.

A binding pocket in receptor TMs and ECLs comprising a total of 35 residues across all valid binding poses makes contact with at least one amino acid within all three dipteran peptides. Ligand-receptor contacts were consistent across all three modeling tools used here: PrankWeb, MDockPep and ClusPro. Notably, the position of contacts between individual peptide and receptor amino acids is conserved even though some of these residues are non-synonymous or chemically dissimilar substitutions across species; this includes *A. aegypti* and *D. melanogaster* as well as *Bombyx mori* PBAN binding to PK2/PBAN-R and *Homo sapiens* NMU/NMS binding to NMUR1 and 2 [85,86,89,97]. Within the binding pocket, the conserved FXPRL peptide motif makes many contacts with conserved TM residues deep within the receptor. The entire peptide interacts with residues at the extracellular surface of the membrane, both in the TMs and ECLs, and a conserved β-turn in ECL2, the latter of which includes a conserved disulfide bond between cysteines in ECL2 and TM3 that is an essential structural feature for Class A GPCR activation [87–89,97]. FXPRLamide N-termini also bind unique amino acids in the ECLs, especially ECLs 2 and 3. Such a pattern of ligand-receptor interactions would allow the functional separation of peptides with unique binding properties and effects without compromising receptor recognition of the shared FXPRL peptide core motif.

### Unique ligand interactions with their receptors provide bases for functional differentiation in *A. aegypti* and ligand-receptor coevolution in *D. melanogaster*

PBAN’s extended 13 amino acid N-terminus interacts with several unique receptor residues, especially in the receptor’s N-terminal domain and distal ECL2 and 3. This would serve to differentiate PBAN binding from that of the much shorter PK2–3 and permit functional divergence of the two peptides. The important role of ECL3 in peptide recognition and receptor activation has been demonstrated in *Heliothis zea*, where replacing the PK2/PBAN-R ECL3 with that of *D. melanogaster* PK1/DH-R and vice versa disrupts ligand recognition as does site-directed mutagenesis in the ECLs [70,90,98]. PK2–3 shares three receptor binding sites with PBAN and has just three unique binding sites, one each in TM3, TM4, and the N-terminal segment of ECL2. Hugin also shares six binding loci with PBAN: five of which are located in ECLs and four comprising non-synonymous amino acid substitutions. ECLs of PK2-R1 are also shorter than those of PK2/PBAN-R, likely reflecting the need to bind just the short hugin peptide which has only 3 N-terminal amino acids compared with 13 in PBAN. The divergence of binding sites and especially ECLs between hugin and PK2-R1 from their homologs PK2–3 and PK2/PBAN-R supports coevolution of peptide and receptor in *D. melanogaster*. Binding of hugin to loci occupied by PBAN and the sparse colocalization of PK2–3 and PBAN binding sites on PK2/PBAN-R suggests that loss of PBAN in *D. melanogaster* removed constraints on hugin binding, permitting interactions with residues in locations that differentiated between PBAN and PK2–3 binding in *A. aegypti*.

Docking models highlight further complexity of receptor-ligand interactions. Individual peptide residues often contact multiple receptor sites, and vice versa. As described previously, peptide N-terminal residues engage unique receptor sites in the ECLs, many of which are non-synonymous between the two species, while the FXPRL core motif anchors the peptide to conserved sites in TMs and proximal ECLs. The variable “X” residue in the FXPRL motif (e.g., FAPRL in PBAN, FSPRL in PK2–3, FKPRL in hugin) has distinct receptor binding interactions in full-length and isolated core motif docking predictions and may provide additional differentiation between peptides binding the same receptor as is the case in *A. aegypti*. Consistent with this prediction, the “X” residue of each peptide is conserved within phylogenetic groups. For example, FAPRL predominates in PBAN sequences of basal Diptera whereas FSPRL appears characteristic of the Lepidoptera [6,36,62]. Future studies will build on these findings by identifying atomic interactions, such as hydrophobic interactions, H-bonds and salt bridges between the bound ligand and its receptor. Increasing resolution of the molecular models and facilitating further comparisons within and across species.

One feature of FXPRLamide neuropeptides that could not be included in the present study is the effect of the C-terminal α- amidation on peptide structure and receptor docking. Functional studies of amidated PRXamide neuropeptides and amidated peptide ligands generally demonstrate that this post-translational modification (PTM) is important for receptor recognition, binding and activation [70,89,93,99–101]. Although online and software tools are capable of modeling some PTMs such as phosphate groups and glycans, none are able to generate models from the peptide sequence that include C-terminal *a*-amidation. These tools include machine learning and LLM-based methods implemented through Python packages, webservers and software packages such as AlphaFold, Rosetta and PyMol [102–105]. Instead, molecular models of amidated peptides are based on structural data generated by NMR, X-ray crystallography or cryo-electron microscopy analyses (for example see [70]) rather than purely *in silico* methods as are used in the present study. The literature suggests that it has been particularly difficult to accurately model post-translational modifications without utilizing experimental data [106–109]. Given the current capacities of the aforementioned *in silico* tools and the prevalence of C-terminal α-amidation in neuro- and endocrine peptides, it is reasonable to expect that modeling of this PTM will soon be possible. Likewise, interactions of the flexible and disordered receptor N-termini have proven difficult to resolve even in X-ray crystallography studies [110]. A recent paper combining multiple approaches finds that N-terminus binding to ligands is transient, a feature that could not be captured in the current *in silico* models [110]. New technologies such as these will continue to develop, narrowing the limitations of molecular docking simulations and enabling a more complete picture of neuropeptide-receptor interactions.

### Predictions of peptide structure suggest a mechanism for functional bias between PK2–3 and PBAN in *A. aegypti* and for ligand-receptor coevolution in *D. melanogaster*

In addition to sequence divergence, differences in peptide secondary and tertiary structure provide a basis for differentiating between similar peptides in *A. aegypti* due to disparate binding and receptor activation properties. Structural models of PBAN consistently adopt rigid α-helical structures in the N-terminal and core motif regions. Docking predictions place the N-terminal along the inner surfaces ECL2 and ECL3 where unique PBAN binding sites are located, which could increase binding affinity and stability of PBAN [111,112]. The core FAPRL motif resides deep in the binding pocket near the base of ECL1, its helical structure likely created due to helix-promoting residues Ala and Pro [95,96,113]. In contrast, PK2–3 lacks a long N-terminus, does not form α-helices and shows greater conformational variability and placement within the receptor in structural models and docking predictions. Binding predictions for hugin to PK2-R1 are similar, suggesting that the compactness and reduced secondary structure of these short peptides might facilitate dynamic binding profiles. In *A. aegypti* in which both PBAN and PK2–3 bind PK2/PBAN-R, peptide structural differences could influence specificity and activation via a mechanism such as ligand bias, in which each ligand elicits distinct conformational changes in the receptor that predispose interaction with different downstream pathways, conferring functional selectivity [114,115]. Functional studies are necessary to test this hypothesis.

### Insights into the roles of core and N-terminal peptide sequences imparted by peptide secondary and tertiary structures

The C-terminal β-turn-forming FXPRLamide core motif alone is sufficient for PK2/PBAN-R and PK2-R activation [79–81]. Interestingly, docking predictions for isolated core motifs in this study did not fully align with the core motifs of full-length peptides. For example, in full-length PBAN, the Phe (F) residue of the core FAPRL motif makes extensive contact with ECL1; in contrast, the isolated core motif is shifted towards the center of the binding pocket, reducing ECL1 engagement. This suggests that although the isolated FXPRL can bind and activate its receptor experimentally, it may have additional interactions in the context of the full peptide. Structural models of the peptides also reveal a surprising feature of peptide tertiary structure that impacts peptide-receptor interactions; a “proline kink” made up of two Pro (P) residues in PBAN and one each in PK2–3 and hugin located between the N-terminus and core motif. This kink in the peptide backbone directs the peptide N-terminus through the center of the receptor. A proline kink is frequently associated with α-helices, producing a bend in the helix that facilitates packing with other helices in transmembrane proteins for example [95,113]. Forcing alignment of docked core motifs of a full PBAN peptide to an isolated FAPRL peptide redirects the N-terminal helix orthogonally, clashing with the ECLs of the receptor. This further suggests that while the FXPRL motif is a key component of peptide recognition and receptor activation, it may operate *in vivo* in a broader context resulting from the influence of the full peptide secondary and tertiary structure on core sequence orientation in the binding pocket. Functional and additional molecular modeling studies will be needed to test whether isolated and full peptide core sequences differ in orientation during binding and in receptor binding and activation properties.

## Supporting information

S1 Table Percent identity of PK2/PBAN-R and PK2-R1 GPCRs by receptor domain. N-terminal extracellular region- EC N term; transmembrane regions- TM 1–7; intracellular loops; ICL 1–3, extracellular loops; ECL 1–3, C-terminal intracellular region; IC C term.
(DOCX)

S2 Table Combined contacts between individual peptide amino acids with regions of their cognate receptors. N-terminal- all peptide residues before the C-terminal core motif FXPRL.
(PDF)

S1 Fig Alignment and comparison of full receptor sequences and binding by FXPRLamide neuropeptides. ***A****.aegypti* PK2–3 and PBAN peptides binding to the PBAN receptor, *D. melanogaster* hugin binding to the PK2-R1receptor as determined using ClusPro. *Bombyx mori* PBAN binding to PK2/PBAN-R and *Homo sapiens* NMU/NMS peptides to NMUR1 and NMUR2 [75,76,80]. Ligand binding sites are shaded; underline indicate transmembrane regions of the receptors.
(PDF)

S2 Fig Receptor amino acids bound by the same residue in all three peptides and in both species. Grey- N terminus, magenta- F, blue- X (A, S or K), green- P, red- R, yellow- L.
(PDF)

S3 Fig All valid ClusPro generated docking models using peptide models produced by PepFold4. The receptor ECL1 is removed to provide a clearer view of the peptide. A-G, PBAN to *A. aegypti* PK2/PBAN-R. H-N, PK2–3 to *A. aegypti* PK2/PBAN-R. O-T, hugin to *D. melanogaster* PK2-R1.
(TIF)

S4 Fig Comparison of docking predictions for PK2–3 and hugin isolated core sequences (yellow) and full peptides (purple) before (PK2–3 A, B; hugin E,F) and after forced alignment of the core motif (PK2–3 C, D: hugin G, H). Arrowheads in all figures indicate a proline residue introducing a kink in the peptide backbone. I, H. Consensus of docking predictions for the isolated FXPRL and full peptide core motifs with their cognate receptors. G. PK2–3 with PK2/PBAN-R, H. hugin with PK2-R1. Colors indicate residues of each peptide: magenta, Phe (F); blue, A; green, Pro (P); red, Arg (R); yellow, Leu (L); grey, N terminal (full PBAN only). Only receptor ECL and TM binding regions are shown.
(TIF)

S1 File Structural models. (ZIP)
