# Supplementary material for: In silico structural and docking models of dipteran FXPRLamide neuropeptides support ligand-receptor coevolution and suggest mechanisms for ligand bias
Source: PLoS One. 2025 Dec 29;20(12):e0329924. doi: 10.1371/journal.pone.0329924 (PMC12747404; doi:10.1371/journal.pone.0329924)
Supplement: S1 Table — N-terminal extracellular region- EC N term; transmembrane regions- TM 1–7; intracellular loops; ICL 1–3, extracellular loops; ECL 1–3, C-terminal intracellular region; IC C term. (DOCX) [file pone.0329924.s001.docx]

**S1 Table**

|  | EC N term | TM1 | ICL1 | TM2 | ECL1 | TM3 | ICL2 | TM4 | ECL2 | TM5 | ICL3 | TM6 | ECL3 | TM7 | IC C term |
| --- | --- | --- | --- | --- | --- | --- | --- | --- | --- | --- | --- | --- | --- | --- | --- |
| % identity | 18.3 | 46.4 | 63.6 | 68.4 | 54.6 | 63.3 | 95.0 | 71.4 | 36.0 | 76.2 | 34.5 | 79.0 | 44.0 | 79.2 | 23.4 |
