# Supplementary material for: In silico structural and docking models of dipteran FXPRLamide neuropeptides support ligand-receptor coevolution and suggest mechanisms for ligand bias
Source: PLoS One. 2025 Dec 29;20(12):e0329924. doi: 10.1371/journal.pone.0329924 (PMC12747404; doi:10.1371/journal.pone.0329924)
Supplement: S2 Table — N-terminal- all peptide residues before the C-terminal core motif FXPRL. (PDF) [file pone.0329924.s002.pdf]

S4 Table

	N-terminus/TM1	TM2/ECL1/TM3	ECL2	TM6/ECL3/TM7	TOTAL
**PBAN**					
F	1	9	1	5	16
A	-	2	4	4	10
P	-	5	4	3	12
R	-	11	2	6	19
L	-	9	4	4	17
N-terminus	5	8	10	18	41
**PK2-3**					
F	-	6	7	6	19
S	-	1	4	2	7
P	-	8	4	9	21
R	-	10	3	5	18
L	-	7	4	4	15
N-terminus	-	9	9	12	30
**Hugin**					
F	-	5	8	7	20
K	-	4	2	9	15
P	-	5	5	5	15
R	1	10	3	7	21
L	1	6	5	9	21
N-terminus	2	8	6	9	25
