# Supplementary material for: In silico structural and docking models of dipteran FXPRLamide neuropeptides support ligand-receptor coevolution and suggest mechanisms for ligand bias
Source: PLoS One. 2025 Dec 29;20(12):e0329924. doi: 10.1371/journal.pone.0329924 (PMC12747404; doi:10.1371/journal.pone.0329924)
Supplement: S1 Fig — A.aegypti PK2–3 and PBAN peptides binding to the PBAN receptor, D. melanogaster hugin binding to the PK2-R1receptor as determined using ClusPro. Bombyx mori PBAN binding to PK2/PBAN-R and Homo sapiens NMU/NMS peptides to NMUR1 and NMUR2 [75,76,80]. Ligand binding sites are shaded; underline indicate transmembrane regions of the receptors. (PDF) [file pone.0329924.s003.pdf]

S2 Figure

```
A.aeg PBAN          -----MMELQQVSTDGGTIVNRSLIYLAMVTAATSSSFVTATDGDAPGVDGFTTRNG-TG      54
A.aeg PK2-3         -----MMELQQVSTDGGTIVNRSLIYLAMVTAATSSSFVTATDGDAPGVDGFTTRNG-TG      54
D.mel Hugin         MLQGVAITIANDSNDDG--INQS--FMAHVSPSPN---QSPSIGVGIGIASSTMANPSES      53
B.mor PBAN          ---------------------------------------------------MMAD--ET       6
H.sap NMUR1 You     -----------------------------MTPLCLNCSVLPGDLYPGGARNPMACNGSAA      31
H.sap NMUR2You      ---------------------------------------------MSGMEKLQNASWIYQ      15
H.sap NMUR2Zhao     ---------------------------------------------MSGMEKLQNASWIYQ      15
                                                                   .

A.aeg PBAN          VDV--------------LLQLP--AVVSINGST-GNGEVMNENFGTKADSICIILPITIF      97
A.aeg PK2-3         VDV--------------LLQLP--AVVSINGST-GNGEVMNENFGTKADSICIILPITIF      97
D.mel Hugin         PEMLLLKNDKFLTHVAHLLNITTENLSNLLGSTNGTNASTMAADSPVDESLTLRTALTVC      113
B.mor PBAN          VNM----------------EMLEN---NLLNVTNVTDQS---SAYSESYPLHLLVPLSVT      44
H.sap NMUR1You      R--------GH--------FDPE-----DLNL---TDE-ALRLKYLGPQQTELFMPICAT      66
H.sap NMUR2You      Q--------KL--------EDPF---QKHLNS---TE--EYLAFLCGPRRSHFFLPVSVV      51
H.sap NMUR2Zhao     Q--------KL--------EDPF---QKHLNS---TE--EYLAFLCGPRRSHFFLPVSVV      51
                                         .    .                :   :

A.aeg PBAN          YCFIFVVGIVGNLAICIVIAKNRSMHTATNYYLFNLAVSDFLLLLFGMPLEVYGTWYPYA      157
A.aeg PK2-3         YCFIFVVGIVGNLAICIVIAKNRSMHTATNYYLFNLAVSDFLLLLFGMPLEVYGTWYPYA      157
D.mel Hugin         YALIFVAGVLGNLITCIVISRNNFMHTATNFYLFNLAVSDLILLVSGIPQELYNLWYPDM      173
B.mor PBAN          YAVIFIVGILGNTSTCVVIARNRSMHTATNFYLFSLAISDIILLVCGLPLELYRLWNPFT      104
H.sap NMUR1You      YLLIFVVGAVGNGLTCLVILRHKAMRTPTNYYLFSLAVSDLLVLLVGLPLELYEMWHNYP      126
H.sap NMUR2You      YVPIFVVGVIGNVLVCLVILQHQAMKTPTNYYLFSLAVSDLLVLLLGMPLEVYEMWRNYP      111
H.sap NMUR2Zhao     YVPIFVVGVIGNVLVCLVILQHQAMKTPTNYYLFSLAVSDLLVLLLGMPLEVYEMWRNYP      111
                    *  **:.* :**   *:** ::. *:* **:***.**:**:::*: *:* *:*  *

A.aeg PBAN          YPFNQVACIITGLLSETATNATVLTITSFTVERYIAICHPFRSHTMSKLSRAIKFVIAIW      217
A.aeg PK2-3         YPFNQVACIITGLLSETATNATVLTITSFTVERYIAICHPFRSHTMSKLSRAIKFVIAIW      217
D.mel Hugin         YPFTDAMCIMGSVLSEMAANATVLTITAFTVERYIAICHPFRQHTMSKLSRAIKFIFAIW      233
B.mor PBAN          YPLGEAQCITIGLASETSANATVLTITAFTMERYIAICRPFMSHTMSKLSRAVRFIIAIW      164
H.sap NMUR1You      FLLGVGGCYFRTLLFEMVCLASVLNVTALSVERYVAVVHPLQARSMVTRAHVRRVLGAVW      186
H.sap NMUR2You      FLFGPVGCYFKTALFETVCFASILSITTVSVERYVAILHPFRAKLQSTRRRALRILGIVW      171
H.sap NMUR2Zhao     FLFGPVGCYFKTALFETVCFASILSITTVSVERYVAILHPFRAKLQSTRRRALRILGIVW      171
                    : :    *       *    *::*.:*:.::***:*: :*:  :   .  :. :.:  :*

A.aeg PBAN          LVAFGLATPQALQFGVVESAH--------TRLCTIKNR--HFEHAFEVSSFLFFVGPMTV      267
A.aeg PK2-3         LVAFGLATPQALQFGVVESAH--------TRLCTIKNR--HFEHAFEVSSFLFFVGPMTV      267
D.mel Hugin         LAAFLLALPQAMQFSVVYQNE--------GYSCTMEND--FYAHVFAVSGFIFFGGPMTA      283
B.mor PBAN          VFALCTAVPQAMQFGIVSYVE----NGQSMSACTVKGP--GVHQVFVISSFVFFVVPMSV      218
H.sap NMUR1You      GLAMLCSLPNTSLHGIRQLHVPCRGPVPDSAVCMLVRPRALYNMVVQTTALLFFCLPMAI      246
H.sap NMUR2You      GFSVLFSLPNTSIHGIKFHYFPNGSLVPGSATCTVIKPMWIYNFIIQVTSFLFYLLPMTV      231
H.sap NMUR2Zhao     GFSVLFSLPNTSIHGIKFHYFPNGSLVPGSATCTVIKPMWIYNFIIQVTSFLFYLLPMTV      231
                      :.  : *::  ..:               * :          .  :.::*:  **:
```

```
A.aeg PBAN          IAVLYVLIGIKLRKSKLLQGVKRQGASIGSGANGIHHHGGGMTTSGGGAGFRSVSGQTRV    327
A.aeg PK2-3         IAVLYVLIGIKLRKSKLLQGVKRQGASIGSGANGIHHHGGGMTTSGGGAGFRSVSGQTRV    327
D.mel Hugin         ICVLYVLIGVKLKRSRLLQSLPRRTFDANR----------------------GLNAQGRV    321
B.mor PBAN          ISVLYALIGLKLRTSRILHPVKKLSLDSNE-------------RPGAHTPYRNGSSQRRV    265
H.sap NMUR1You      MSVLYLLIGLRLRRERLLLMQEAKGRGSAAARSRY-----------TCRLQQHDRGRRQV    295
H.sap NMUR2You      ISVLYYLMALRLKKDKSLEADEGNAN-------------------------IQRPCRKSV    266
H.sap NMUR2Zhao     ISVLYYLMALRLKKDKSLEADEGNAN-------------------------IQRPCRKSV    266
                    :.*** *:.::*: .: *                                      :  *

A.aeg PBAN          IRMLVAVVATFFFCWAPFHAQRLMAVYGAVTNT---DNEFFFQVYTYLTYISGILYFLST    384
A.aeg PK2-3         IRMLVAVVATFFFCWAPFHAQRLMAVYGAVTNT---DNEFFFQVYTYLTYISGILYFLST    384
D.mel Hugin         IRMLVAVAVAFFLCWAPFHAQRLMAVYGLNLINIGISRDAFNDYFRILDYTSGVLYFLST    381
B.mor PBAN          IRMLVAVALSFFICWAPFHVQRLLAIYGKSLEH---PSDTFYLVYIVLTFLSGVLYFLST    322
H.sap NMUR1You      TKMLFVLVVVFGICWAPFHADRVMWSVVSQ------WTDGLHLAFQHVHVISGIFFYLGS    349
H.sap NMUR2You      NKMLFVLVLVFAICWAPFHIDRLFFSFVEE------WSESLAAVFNLVHVVSGVFFYLSS    320
H.sap NMUR2Zhao     NKMLFVLVLVFAICWAPFHIDRLFFSFVEE------WSESLAAVFNLVHVVSGVFFYLSS    320
                     :**..:.  * :****** :*::              : :   :  :   **::::*.:

A.aeg PBAN          CINPLLYHIMSHKFRDASRRTLKLGCCGIDSKR---SDAQNHTYSALSRYGITGN----G    437
A.aeg PK2-3         CINPLLYHIMSHKFRDASRRTLKLGCCGIDSKR---SDAQNHTYSALSRYGITGN----G    437
D.mel Hugin         CINPLLYNIMSHKFREAFKITLTRQFGLARNHHHQQSQHHQHNYSALLRQNGSMRLQPAS    441
B.mor PBAN          AINPFLYNIMSNKFRNAFKMTLAAWCGRRGGPR------MGRSYSALLASQRQRA-----    371
H.sap NMUR1You      AANPVLYSLMSSRFRETFQEALCLGACCHRLR----PRHSSHSLSRMT--TG-----STL    398
H.sap NMUR2You      AVNPIIYNLLSRRFQAAFQNVISSFHK--QWH----SQHDP-QLPPAQRNIF-----LTE    368
H.sap NMUR2Zhao     AVNPIIYNLLSRRFQAAFQNVISSFHK--QWH----SQHDP-QLPPAQRNIF-----LTE    368
                    . **.:* ::* :*: : : .:

A.aeg PBAN          GSFKTGANAQYGTPVAQNRPSTDLQFCQQESEMSLLRSEPGRCLKSETHCVSI---SSQS    494
A.aeg PK2-3         GSFKTGANAQYGTPVAQNRPSTDLQFCQQESEMSLLRSEPGRCLKSETHCVSI---SSQS    494
D.mel Hugin         CSVNNNALEPYGSY-RV---------------------VQFRCRDANHQLSLQ---DSIR    476
B.mor PBAN          ---ANGLTDPVRGPRRL---------------------RRLS-TATTHLCDAP---PRAQ    403
H.sap NMUR1You      CDVG--SLGSWVHPLAGND---G---------------PEAQQETD------P-------    425
H.sap NMUR2You      CHFV--EL-------TEDI---G---------------PQFPCQSSMHNSHLPAALSSEQ    401
H.sap NMUR2Zhao     CHFV--EL-------TEDI---G---------------PQFPCQSSMHNSHLPAALSSEQ    401

A.aeg PBAN          TIITT---LSHSGTTRSSSIERKQ--PNGRPVYRTSGSFG--------------------    529
A.aeg PK2-3         TIITT---LSHSGTTRSSSIERKQ--PNGRPVYRTSGSFG--------------------    529
D.mel Hugin         TTTTTTT-INSNSMAAGNGVGGGAGGGGGGRRLRKQELYGPGPGTAVPHRMLQAQVSQLS    535
B.mor PBAN          VSATKIA-ISP-------------------------------------------------    413
H.sap NMUR1You      -S----------------------------------------------------------    426
H.sap NMUR2You      MSRTNYQSFHFNKT----------------------------------------------    415
H.sap NMUR2Zhao     MSRTNYQSFHFNKT----------------------------------------------    415

A.aeg PBAN          ---SRGSIR--------------------------TGVPLENGDP------GGRLSTIAE    554
A.aeg PK2-3         ---SRGSIR--------------------------TGVPLENGDP------GGRLSTIAE    554
D.mel Hugin         SLGDANSLLEAEVVDRHYASGRAKRALLATKSGALLVTPPQSGDPSEVSQPATRLKLTRV    595
B.mor PBAN          ------------------------------------------------------------    413
H.sap NMUR1You      ------------------------------------------------------------    426
H.sap NMUR2You      ------------------------------------------------------------    415
H.sap NMUR2Zhao     ------------------------------------------------------------    415

A.aeg PBAN          KLRRGTKKVLQFSKSPSTSPTKTSTGSVSAPD------------RDAKRRWLRKKESVDS    602
```

```
A.aeg PK2-3       KLRRGTKKVLQFSKSPSTSPTKTSTGSVSAPD------------RDAKRRWLRKKESVDS     602
D.mel Hugin       ISRRD--EVAN-------TSTPPFCGSHSLPDPETCQSASVAGRSSRKFPWRKRRQKTED     646
B.mor PBAN        ------------------------------------------------------------     413
H.sap NMUR1You    ------------------------------------------------------------     426
H.sap NMUR2You    ------------------------------------------------------------     415
H.sap NMUR2Zhao   ------------------------------------------------------------     415

A.aeg PBAN        VDTNTISNSSLKEYDEEEFSSAELAKFMAEINNEIR      638
A.aeg PK2-3       VDTNTISNSSLKEYDEEEFSSAELAKFMAEINNEIR      638
D.mel Hugin       PSSEGLTYGSPKSQ----------------------      660
B.mor PBAN        ------------------------------------      413
H.sap NMUR1You    ------------------------------------      426
H.sap NMUR2You    ------------------------------------      415
H.sap NMUR2Zhao   ------------------------------------      415
```
