# Supplementary figures and images for: In silico structural and docking models of dipteran FXPRLamide neuropeptides support ligand-receptor coevolution and suggest mechanisms for ligand bias
Source: PLoS One. 2025 Dec 29;20(12):e0329924. doi: 10.1371/journal.pone.0329924 (PMC12747404; doi:10.1371/journal.pone.0329924)

S3 Figure

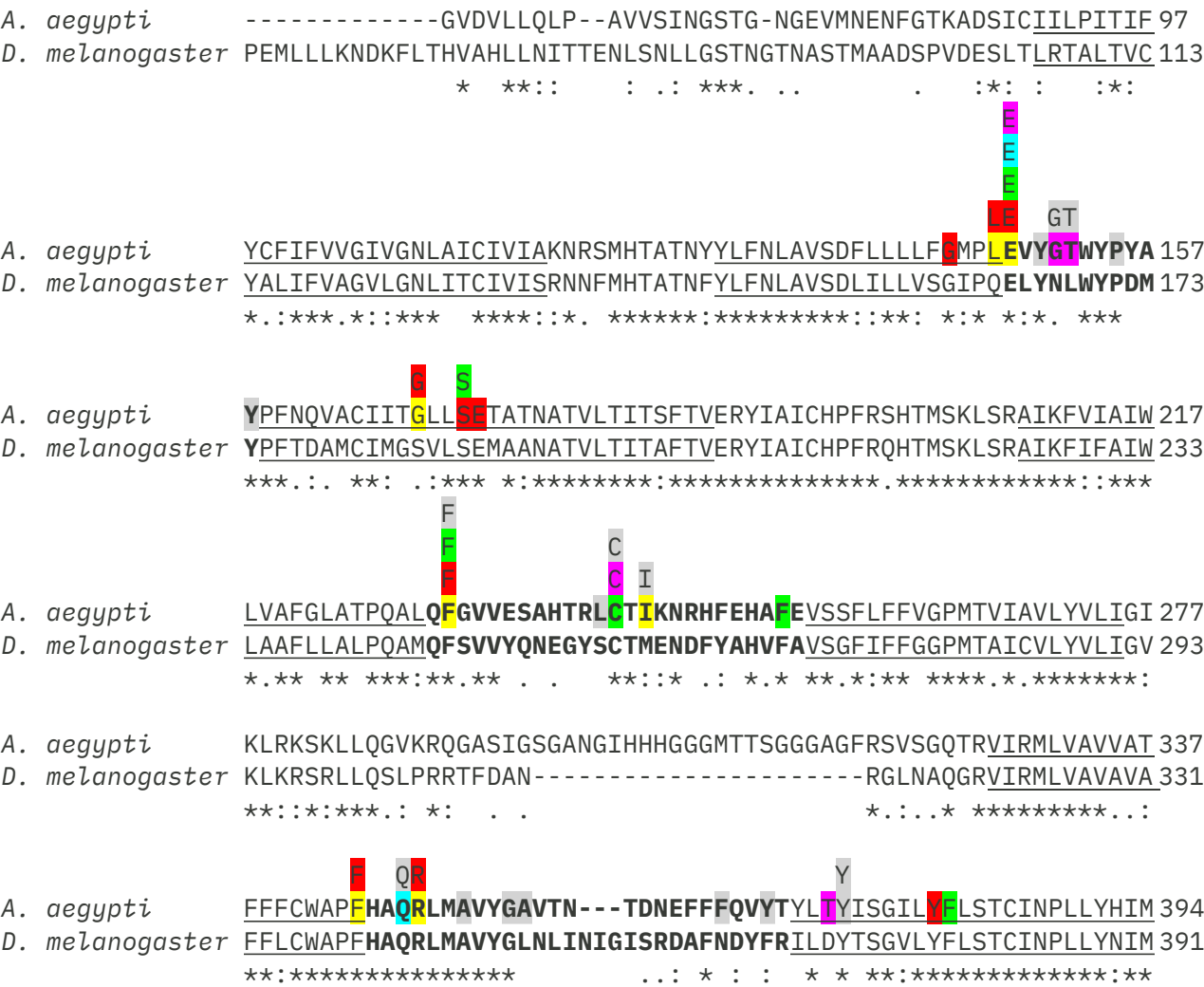

Supplement: S2 Fig — Grey- N terminus, magenta- F, blue- X (A, S or K), green- P, red- R, yellow- L. (PDF) [file pone.0329924.s004.pdf]

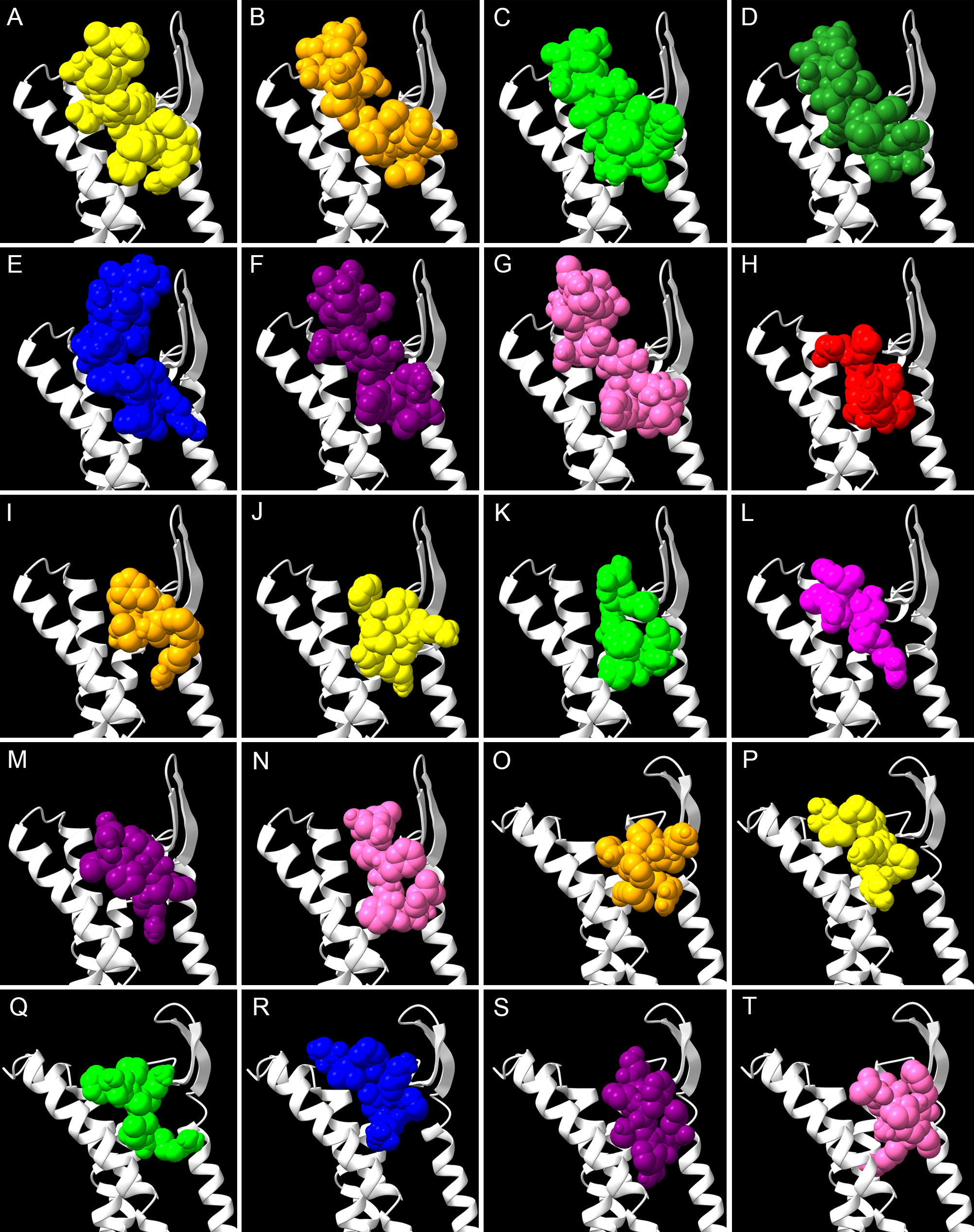

Supplement: S3 Fig — The receptor ECL1 is removed to provide a clearer view of the peptide. A-G, PBAN to A. aegypti PK2/PBAN-R. H-N, PK2–3 to A. aegypti PK2/PBAN-R. O-T, hugin to D. melanogaster PK2-R1. (TIF) [file pone.0329924.s005.tif]

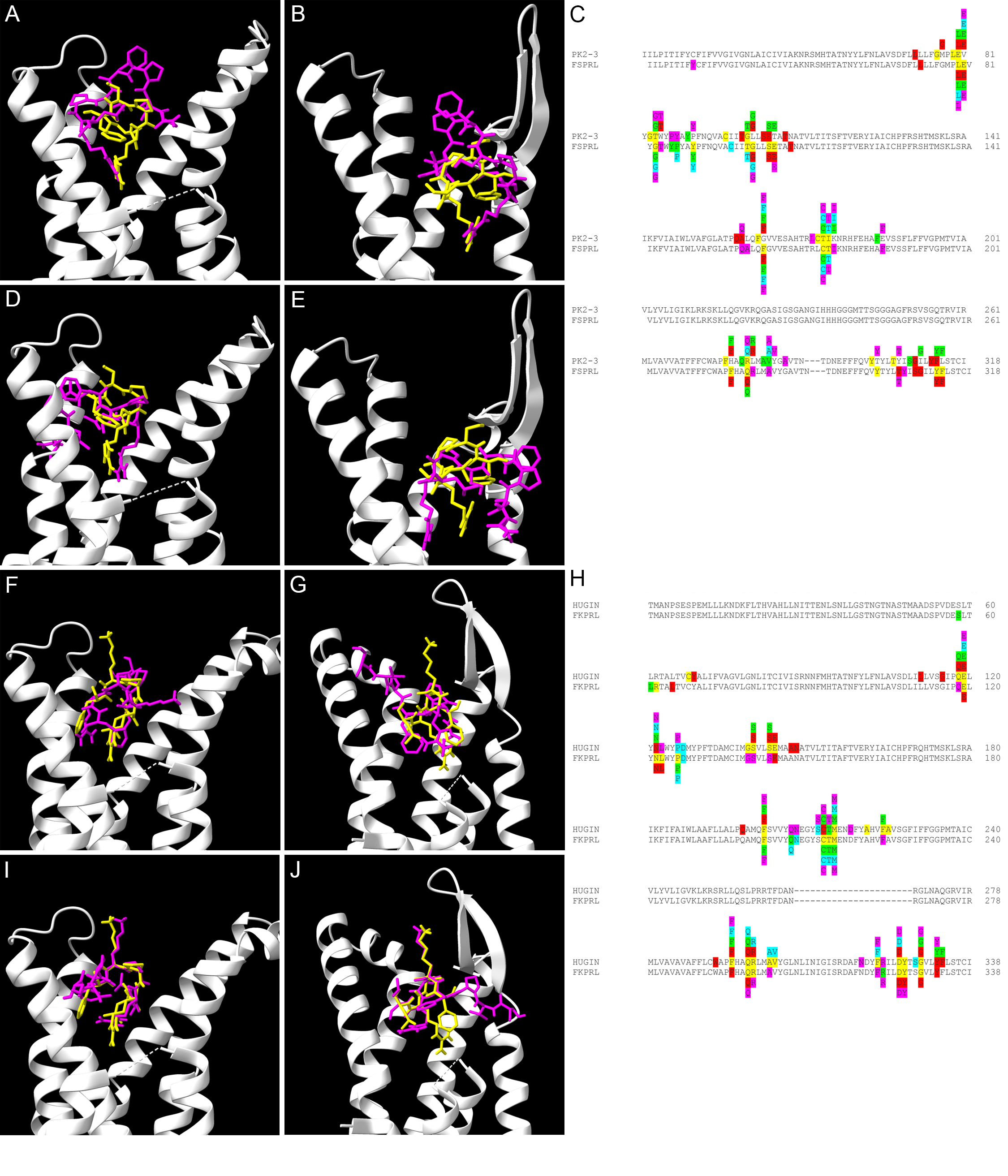

Supplement: S4 Fig — Arrowheads in all figures indicate a proline residue introducing a kink in the peptide backbone. I, H. Consensus of docking predictions for the isolated FXPRL and full peptide core motifs with their cognate receptors. G. PK2–3 with PK2/PBAN-R, H. hugin with PK2-R1. Colors indicate residues of each peptide: magenta, Phe (F); blue, A; green, Pro (P); red, Arg (R); yellow, Leu (L); grey, N terminal (full PBAN only). Only receptor ECL and TM binding regions are shown. (TIF) [file pone.0329924.s006.tif]
